# Cellular abnormalities in Autosomal Dominant Polycystic Kidney Disease (ADPKD) fibroblasts

**DOI:** 10.1186/2046-2530-1-S1-P95

**Published:** 2012-11-16

**Authors:** S Moyes, J Norman, PD Wilson

**Affiliations:** 1 UCL Centre for Nephrology, UCL Medical School, London, UK

## 

ADPKD is a common, monogenic disease in which aberrant polycystin (PC)-1 function leads to end-stage renal disease. Progressive tubular epithelial cyst enlargement is accompanied by variable amounts of tubulointerstitial fibrosis contributing to disease progression. Although ADPKD abnormalities have been studied in detail in cystic epithelia where PC-1 is localised in apical cilia and basal matrix-associated focal adhesions (FA), little is known about PC-1 or cilia in ADPKD fibroblasts. Previously we identified a hyperproliferation defect in ADPKD fibroblasts analogous to cystic epithelia. Here we use matrix-adhesion, Western blot and immunolocalisation in human “early”-stage, pre-dialysis (E-), and end-stage (ES-) ADPKD versus normal (N) tissues and cells to further characterise differences between these fibroblasts. *In vivo*, stage-dependent increases in αSMA-containing fibroblasts, interstitial collagen deposition and cystic-epithelial TGFβ content were seen in ADPKD kidneys together with focal interstitial PC-1 expression. *In vitro* primary cultures of N, E- and ES-ADPKD fibroblasts showed stage-related increases in matrix adhesion; increased spreading; upregulation and phosphorylation of FA proteins, FAK and paxillin. αSMA was absent in N but markedly upregulated in ES-ADPKD fibroblasts and incorporated into stress fibres. All types of fibroblasts produced cilia; cilia length decreased with disease stage. Fibroblasts also expressed PC-1 but only N expressed full-length PC-1 (460kD) and the ~250kD fragment. Several smaller fragments (30-100kD) were detected in ADPKD, mostly decreasing with disease stage. Interestingly, the putative C-terminal ~30kD PC-1 fragment was downregulated in ADPKD fibroblasts which also showed reduced nuclear PC-1 staining. We conclude that abnormalities in PC-1 and cilia may play important roles in the ADPKD fibroblast phenotype.

